# Service Innovation, Inter-organizational Trust, and Performance in Hospital Platforms: Social Network and Agency Perspectives

**DOI:** 10.3389/fpubh.2022.819371

**Published:** 2022-03-17

**Authors:** Jianhong He, Chenxu Hu, Chih-Chen Lin

**Affiliations:** ^1^School of Economics and Management, Chongqing University of Posts and Telecommunications, Chongqing, China; ^2^Postgraduate Program in Management, I-Shou University, Kaohsiung, Taiwan

**Keywords:** service innovation, inter-organizational trust, service performance, organizational agency, social network, hospital

## Abstract

Service industries contribute significantly to the economic, social, and even life aspect of the world. However, service innovation has been rarely discussed in healthcare context, especially in the digital healthcare context Service innovation needs to be organized in the premise of mutual trust to be efficient, thereby improving service performance. The trust and efficiency here demands a good online platform service to both virtualize the interaction processes and maintain trust and agency. This research uses social network theory and agency theory to emphasize the importance of trust in cooperation in hospitals, and the relationship between organizational trust and organizational performance. Furthermore, we analyzed the role of agents in enhancing the relationship between service innovation and trust. Based on the analyses, five propositions and future research directions are proposed.

## Introduction

Service innovation has become an important key and means for hospitals to compete with each other and succeed. However, service innovation has less been discussed in the context of digital platform in healthcare organizations, especially in hospitals' extended inter-organizational relationships. Nowadays, digital platforms serve as a non-human bridging and bonding mechanism for large-scope, cross-boundary operations and collaborations. A hospital can be treated as one open, interconnected platform-based organization because the service delivery for value creation depends largely on the intense interactions between the personnel of a hospital, the patients and families, government, community, etc. In such context, a digital platform can assist in virtualizing such interactions and value co-creation. Thus, it is critical to discuss the inter-organizational relationships in the context of hospital service innovation.

In the case of competition between various platforms, network service innovation has promoted transactions and controlled the hospital's connection due to network strategies. This is also true in a healthcare organization, which simultaneously contains non-for-profit service spirits and public service agency, as well as money transactions at the same time. The transaction competition between two or more different users connected through indirect networks has attracted attention from all walks of life. The bilateral market of the network has evolved into the current multilateral platform, which is dependent on sales, execution, and knowledge sharing (1). How organizations create new network service value has received less attention in service innovation research in the early stage, and focused on the results and process of service innovation (2). The research on service innovation allows us to understand the types and strategies of service innovation and so on. Most innovation research focuses on products rather than services. (3). The strategic plans established for the development of new products mostly focus on profitability. Innovation is a necessary choice for the industry, not just a strategy (4). Product development is the basic process for the success and survival of organizational innovation. Service innovation is an important link for organizational structure and hospital progress. Service innovation is divided into tangible and intangible. The development of intangible products for new services is an activity for the benefit of customers, while tangible products are the concept of product manufacturing innovation (5).

The process of service innovation development is different from the process of product development in some respects. Past studies have regarded the service innovation process as the service process of product technology innovation. Service innovation is not a new concept, but in innovation research, most scholars focus on the research of technological innovation in manufacturing organizations (6). After decades of continuous research by many scholars, different views on current service innovation have gradually formed, such as assimilation, differentiation, and integration. So far, most studies on service innovation have been discussing product-related logic (7). According to Schumpeter's economic growth theory. It is divided into different types of innovation, such as: product, process and organizational innovation. The value of innovation is not defined by the output of the hospital, but by how the hospital provides better services.

In addition, the past research seldom discussed network service innovation from the perspective of agency theory. The service innovation of close interaction with customers highlights the importance of external relationships, and involves the exchange of knowledge between hospital interactions. Early research on service innovation focused more on the innovation within the hospital, while ignoring the key service innovation in the external partnership structure. In recent years, some scholars have explored the research of open innovation, and have been focusing on the resources obtained by the internal development of the organization and external partnerships. External development is especially important for the acquisition of knowledge. The combination of stakeholder relations is the result of all the combination stakeholder relations of the organization, showing that the allocation and management of the investment portfolio affects service innovation (8). Enterprise Services stakeholder relations with external parties to achieve innovation goals. This is usually because they do not have the resources they need, and lack the ability to develop the resources they need internally. Or through the relationship between partners to achieve complementary goals for external resource cooperation.

Therefore, the purpose of this article is to analyze how agency relationships regulate the impact of network service innovation on organizational performance, and explore from the perspective of agency theory, whether inconsistencies in agency relationships will have a negative impact on organizational performance. The research question explores how the agency relationship regulates the impact of network service innovation on organizational performance. The difference of the inconsistency scale of agency theory relationship represents the inconsistency of goals. Will it affect the service performance of the hospital?

## Theoretical Basis, Literature Review and Discussion

### Theoretical Basis: Organizational Trust, Agency Theory, Social Capital

#### Organizational Trust and Social Capital

Inter-organizational trust is the antecedent of establishing exchange relationships between organizations. Since the organization is not an individual, and its thinking direction prioritizes interests, previous studies believe that this mode of thinking is a consideration of (calculative trust). In the (inter-organizational context), the degree to which computational trust will become the core of the exchange relationship will affect the actions taken by both parties in this relationship. When computational trust accounts for a greater degree, “writing a contract” is a way to ensure that both parties to the exchange perform their responsibilities and obligations, and avoid possible risks through “prevention”. Previous research called it “bounded rationality” (9). Therefore, the establishment of a contractual relationship is based on the position of “dis-trust carefully” (10). Through the contract, the two parties in the exchange relationship are expected to exchange their expectations Keep under the same cognitive framework to maintain the cognitive consistency of the exchange relationship between the two parties. When the exchange relationship does not develop as expected (i.e., the consistency of cognition is broken), since the contract has established a default range acceptable to both exchange parties, both parties only need to make corresponding adjustments to respond to the default (11).

Another type of trust is presumptive trust. Presumptive trust refers to the organization's characteristics, identities, roles, rules and other information, which can help exchange parties to establish positive social expectation (12), and then promote trust. In other words, presumptive trust builds trust on “social identification.” Since the presumed trust between the organization and the organization must be impersonal, the social identification information of the organization is aggregated from the main characteristics of the members of the organization. When the social identification information between the organization and the organization is more similar, the presumed trust between the two parties is stronger (11). At the same time, the leader of the organization can determine the appearance of the organization, and the display of the leader's leadership will also be presented in the organization's social information. For example, leaders are very particular about product quality, and the organization's social identification information may be reflected in the organization's good reputation for products. This kind of trust relationship is established on the fact that both organizations can clearly recognize the position, responsibilities and obligations of their work roles, and take actions that match the roles, so as to maintain the consistency of social identification of each organization, so that they can exchange relationships. Establish a presumptive trust relationship.

In addition, the trust relationship between the leader and the leader can also be inferred to the relationship between the organization and the organization. When the leader of an organization has a good relationship with other leaders, the leader can identify opportunities through personal social networks (13), obtain resources (14), and mobilize Resources (15) and establish legitimacy for its organization (16). From the perspective of Social Capital, an excellent social network can help organizations achieve better performance. There are several key points in the transformation of social networks into social capital: one is structural hole, the second is the strength of ties, and the third is network diversity. The former focuses on whether the organization occupies the main node of the network, the two discuss the strength of connection with other organizations, and the last is the degree of tightness of the network structure. The three can determine the advantages and disadvantages of the organization in the entire social network, and then enable the organization to use the network to occupy a part of the industrial chain.

In an online healthcare communication context, there is a partnership between hospitals, or cooperation between projects, which needs to be shared or authorized among multiple hospitals. In the environment of multiple networks, it may be necessary to exchange and share resources between different domains. Therefore, the exchange between multiple domains involves each hospital performing its duties and building bridges, so the trust and connection between hospitals is very important. At the same time, through the exchange of resources between hospitals, the online environment is promoted to be effective. Therefore, this research analyzes from the perspective of social capital and organizational trust.

#### Agency Theory

Agency relationship is a kind of contract. According to the contract, one or more people (principal) hire another (agent) to provide certain services on their behalf, including entrusting certain decision-making powers to the agent, according to the scholar (17) proposed agency theory. This theory later evolved into the theory of signing a contract as a transaction behavior. The hospital is composed of a series of contracts. The supplier holding capital and the management department of the operation, the hospital and the supplier (customer), and the hospital and the employee are a series of contractual relationships. The agency's operating power is mainly the contractual relationship between the organization's resources given to specific suppliers. The relationship between the organization's shareholders and managers conforms to the agency relationship. According to the agency theory, the hospital entrusts the management right to the manager's agent, and then delivers it to the manager in the form of a contract. Including capital providers and capital operators.

Agency theory is mainly the relationship between the two parties' commitment to the contract. The contractual relationship between the provider and user of corporate capital. When the manager himself is the holder of corporate capital, the manager is working hard for his own hospital. Under this circumstance, there is no so-called agency problem. If the hospital obtains capital through debt, not through the hospital's issuance of stocks, there is also the agency problem, but the method of obtaining it is different (17).

The agent has more information about the business operation than the principal. In the case of two principals, these principals may have different risk preferences. If the interests are inconsistent and the goals are inconsistent, then the information may be hidden, resulting in information asymmetry. The relationship between principal and agent operates most efficiently. It can be used to view the explicit (legal) and implicit (social) aspects of the contract. This kind of information asymmetry will affect the principal's effective supervision of the agent manager. It is possible that some of these actions will damage the rights and interests of the principal (business owner) and cannot be monitored in time. For example, under the guise of any name, increase one's wealth. In order to protect the respective interests of both parties and the cooperative relationship, the owner of the hospital and the agent manager will sign a contract. In order to reduce the risk of the agent and protect the interests of the agent, the two parties will sign a contract to protect the rights of both parties. For example, the financial report will be reviewed externally, and the agent managers will perform their duties carefully in order to prove their efforts to the corporate clients.

Agent managers will also ask for the right to exercise internal audits for their own interests, so that the principal can understand the efforts of the agent managers (17). When one (agent) represents another (principal), there is a principal-agent relationship. These relationships are usually because the agent has some professional knowledge. however. Because there is an asymmetric information behavior pattern, it can satisfy the agent's own interests, not the interests of the principal. Agency theory believes that the corporate governance mechanism helps to align the interests of the principal with the interests of the agent. For example: clear instructions, provision of audit standards, some incentive measures, such as variable compensation or bonuses, can indicate that the interests of the agent are aligned with the interests of the principal. In terms of employment, employers (principals) can use piece rate/commissions, profit sharing, efficiency wages, performance measurement (including financial statements), agents to post security deposits or threats of termination of employment, so that the interests of workers are in line with their own interests be consistent. If the interests of the principals often diverge, in addition to the moral hazard that the agents still face, they will also face incentives that promote their personal interests rather than the common interests of all clients. Problems can also arise when organizations increasingly respect powerful management. Since shareholders are deprived of the power of supervision, management problems may also arise.

In the case of two principals, these principals may have different risk appetites. Information is regarded as a commodity that can be exchanged. If the interests are inconsistent and the goals are inconsistent, then the information may be hidden, resulting in information asymmetry. The relationship between principal and agent operates most efficiently. It can be used to view the explicit (legal) and implicit (social) aspects of the contract. It involves solving the measurement and incentive problems that occur when the principal and the agent have different goals and desires, and the principal verifies that the agent's performance is economically.

### The Evolution of Service Innovation

The process of service innovation development is different from the process of product development in some respects. Past studies have regarded the service innovation process as the service process of product technology innovation. Service innovation is not a new concept, but in innovation research, most scholars focus on the research on technological innovation of manufacturing organizations (6). After decades of continuous research by many scholars, different views on current service innovation have gradually formed, such as assimilation, differentiation, and integration. The definition of service innovation has crossed different fields. The current service innovation mainly elaborates on four aspects: First, the result of service innovation is separated from the development process. Second, innovation must be put into action. Third, innovation must be a new concept. Fourth, innovation must create value (18). Service innovation In the manufacturing industry, the innovation process is a common service process for manufacturing and production, and service is a behavior or process rather than a product. In the past ten years, the innovation trend has been to require supplier networks to provide products and services, and to connect with suppliers and customers through technology. This is the concept of application and reorganization innovation.

Since the development of service innovation research, scholars have developed another open innovation research. Most of this research focuses on internal development and externally obtained resources. External partnerships are essential for stakeholder relation integration. Partners are the most critical operating procedures for service innovation. In order to acquire knowledge, hospitals combine with various stakeholder relations, including customers, suppliers, and competitors, to achieve the goal of promoting service innovation. And organizations form stakeholder relations with external parties to achieve the goal of innovative services. This is usually because they do not have the resources they need each other, or they lack the ability to develop the required resources internally. force. Or want to obtain external resources through the relationship between partners to achieve complementary goals.

### Team Network Service Innovation

The innovation of network organization is the products and services provided by the supplier network. In order to take full advantage of this relationship, the product is expanded and developed into a combination of services and products provided by an organization's network. According to Powell (1990), the network of organizations is regarded as a relationship of mutually beneficial and interdependent organizational forms. In a network organization, suppliers or partners provide resources to jointly design or jointly produce innovative services and products.

The network platform refers to the products and services that connect the two groups of users in the bilateral network. The business operation model of the platform has the phenomenon of increasing returns to scale. Under each platform is a layered platform after another. The network The platform is divided into four layers: infrastructure support, reading support, content selection and content, allowing us to better understand the structure of the platform. The emergence of Internet platforms and various emerging technologies has changed the relationship between hospitals and changed the traditional linear structure business model to a diversified and connected market structure, resulting in innovative services. The knowledge sharing provided by the Internet platform and the essential aspects of the innovative services included in the market economy have been transformed from the old thinking and extended, and the participation in the activity planning mechanism highlights the innovation of its differentiated services. In the online platform market, although the innovation driven by product recognition innovation and differentiated services is felt, in the evolution and development of Taiwan's online platform, the innovative services of the online platform have gradually gained the trust of consumers, but still There are many aspects that still need to be overcome one by one. From the perspective of knowledge sharing and innovative services, there is still considerable room for development in providing a consumer-oriented service innovation model. However, for the legal issues that must be paid attention to in the commercial activities of the online platform, such as consumer protection, protection of intellectual property rights, maintenance of transaction security, protection of privacy rights, etc., existing laws must be regulated to provide industry players. There is a good system to follow for the development of online platforms. The Internet platform connects platform operators, hospitals, and consumers to continuously innovate services, and through the experience of mutual cooperation and connection, provides innovative and differentiated services to meet the needs of consumers, and to share and create value. The connection of innovative service models all come from the mutual trust relationship between consumers and hospitals, which is the product of the sharing of knowledge. The emergence of online platforms has brought different thinking directions for business owners and consumers, and found that business owners and consumers produce value together, and develop and pursue value creation together, and rely on mutual trust and reasonable and even distribution. The act of creating added value. The Internet platform is to provide more information services to meet the needs of consumers, and to provide more value through the information management system. The network platform provides a brand new service in the cloud, which makes it more convenient and stable for consumers to use, and overcomes the limitations of mobile devices. The network platform cloud service enables suppliers to expand and support various online services. Enhance many competitive advantages.

The interaction of partners or stakeholder relations is through the mutual exchange of knowledge and complementation, and the management of mutual cooperative relations. The mutual exchange of knowledge among partners is related to the degree to which new knowledge is created through mutual influence. The relationship between partners and stakeholder relations and mutual management is an important key role in service innovation, and mutual trust, learning and sharing of professional knowledge to achieve common goals, and active participation in activities and execution are very important. While the combination of stakeholder relations becomes partners, they also learn from each other, rely on and enjoy various resources. And by sharing knowledge and co-creating and sharing new product development and services with partners.

An hospital is a system in which there are various important resources, processes and behaviors, and suppliers and partners are important in this role. The network combines the competitiveness of different suppliers with tangible and intangible technologies. The results of this combination of different suppliers are different.

In the stakeholder relation of hospitals, suppliers play an important role in the key to service innovation. In order to obtain the source of knowledge, the hospital forms stakeholder relations with various hospitals to share resources and share risks. It is also because of this stakeholder relation to learn more about new technological developments. And often competitors may also become partners of technology and knowledge sharing, and develop toward a common goal.

In the past 10 years, many suppliers have been carrying out their innovative services. With the rapid development of network technology, the demand for the network is increasing. The rapid growth of the network has caused the continuous development of innovative services. And the innovation of service innovation through network stakeholder relations has created a new situation in the industry, whether it is technology. The growth, the different types of services and the value created cannot be ignored.

Suppliers through stakeholder relations are considered a powerful source of knowledge, because they often learn more about new technological developments, and because of these cooperative stakeholder relations and cooperation to develop new services, they also share investment and risks. On another level, it also has a considerable impact on hospital performance. Past studies have shown that innovation is to treat stakeholder relations as a learning tool for insights generated when managing stakeholder relation relationships. The extent to which customers participate in stakeholder relation cooperation has a differentiated impact on service innovation. Customers provide diversified experience sharing, and organizations can use the shared experiences of customers to use it to improve innovative ideas for future products and services. Differences in the degree of customer participation have different impacts on service innovation. Customer partnership is to learn from each other, have common goals, common responsibilities, mutual trust, and participation to promote service innovation.

### Performance

The network between organizations is a kind of exchange or transaction relationship between organizations. The stakeholder relation between organizations and external suppliers is usually because both parties take what they need and supplement the resources they need, and complement each other through the relationship between the two partners their common goal. Hospitals form stakeholder relations because of their strategies, and the formation of strategic stakeholder relations regards service innovation as a tool for mutual learning between hospitals, focusing on the expansion of product innovation and the development of new services. The network hospital formed through the hospital's external network will improve the existing service process and develop together through the network relationship. If the hospital members have a benign dynamic relationship and tacit understanding, they can help the hospital members to complete their tasks more effectively. The ability to direct the relationship to the goal of the organization, so that the transaction cost between the organization and the manufacturer is greatly reduced, and the organization can produce high results (19).

### Trust

In social sciences, trust is considered a dependency. Trustworthy individuals or groups mean that they abide by ethics, laws, and previous commitments to implement policies. However, trust is to simplify the cooperative relationship between people, whether it is at the interpersonal, inter-team, and inter-organizational levels, there is interdependence. Interdependence means that there is an exchange relationship between the two parties. Regardless of the content of the exchange, it means that the two parties have at least a certain degree of interest. Inter-organizational communication in organizational management is one of the necessary conditions for maintaining the relationship between internal personnel of the organization, and its purpose is to promote mutual trust as objective trust through the interconnection of internal members of the organization.

According to (20), the definition of trust is when one party is willing to be affected by the actions of the other party because it expects that the other party will work hard to implement the tasks assigned by the client without supervision. Or doubt the ability of the other party. Trust itself does not take risks, but the willingness to take risks. The willingness to take risks is one of the few characteristics that all trust funds share. This definition of trust applies to a relationship that is identifiable to another party, and the other identifiable party is considered to respond to and respond to actions taken by the principal. This definition is the same as (21). The definition is similar (22) means that there are some important things to be lost, making yourself vulnerable. This has blurred the nature of trust. These include cooperation, confidence and predictability (23).

### Organization Agent

Ross (24) paid great attention to the development of agency theory. In principle, the agency literature proposes the recommendations of the contract agent between the parties: (1) the preferred structure of the contract parties, (2) the uncertainty of nature, and (3) the information structure in the environment. And usually it is more focused on risk sharing, and the relationship between the principal and the agent in the form of contract (25).

The agency relationship is defined as a contract. According to the mutual agreement of the contract, the connection between one or more (principals) and another (agent) represents that they are implementing certain service agreements, which involves some decision-making powers that will be granted to the agent. If this kind of mutual relationship, both parties are the greatest utility, then there are good reasons to believe that the agent does not always act in the interests of the principal.

The deposit or cost is to ensure the principal to ensure that the agent will not take certain actions that will harm the principal. And get compensation when taking such actions. However, it is usually impossible for the principal or agent to ensure that the agent makes the best decision from the principal's perspective at zero cost (17).

## Analysis and Development of the Relevance Between the Various Constructs

### Team Network Service Innovation and Team Performance

The performance of modern network distribution depends to a large extent on the network services used for information between computers, and the development speed of these services is much lower than the environment established by computer computing systems. The slow evolution is neither due to a lack of demand nor a lack of innovative ideas, but because of the changes in various computer network connections that do not support multimedia applications and accommodate more potential mobile hosts, although the requirements for these changes were reached a long time ago. They agree, but they have not yet fully deployed. The main problem is that the current process of changing network protocols is lengthy and difficult. Therefore, the requirements for standardization should be based on network protocols and interactive operability, which means that there must be a clear time and consensus on requirements. Once the new agreement is accepted, it can be completed in a compatible and renewed manner. Therefore, through the innovative method of network service, it can be a standardized communication model instead of a separate communication protocol. The potential resources provided by the network platform are difficult for the industry to access through their own marketing methods. For example: OTA can help hotels protect and Process reservations, communicate with guests and manage reviews. OTA can quickly respond to government policies, integrate resources, and create service value together. OTA can protect the profit of the hotel and not become a price war. Therefore, the strength of ties can improve work performance under the efficient use and execution of the network (26).

#### Proposition 1

The strength of the network connection will enhance the team's work performance under the efficient use of network services.

### Team Network Service Innovation and Team Trust

Service has become a key value driver for each hospital. Currently, there is a lack of understanding of the science on which the design and operation of service systems depend. New conceptual understanding and theoretical foundation are needed to systematically describe the nature and behavior of the service system. Therefore, the mobile network theory can be used as a theoretical perspective to study the development and adoption of service innovation. The development and adoption of service innovation requires the integration of multiple elements across hospitals, including people, technology, and networks. In order to succeed in service innovation, it is necessary to coordinate and coordinate the technologies and interests of actors. Therefore, as an understanding of the relationship between actors, and to show the development and adoption of service innovation of these actors in various organizations to meet their needs through network formation, it will definitely show the expected goal of service innovation and development, and be able to win the trust Service object (27). When the leader of an hospital has a good relationship with other leaders, the leader can obtain resources through the personal social network (14), and the trust relationship between the leader and the leader can also be inferred to the hospital and the relationship between the hospital. Presumptive trust (presumptive trust) is through the hospital's characteristics, identities, roles, rules and other information, this information can help exchange parties to establish a positive social expectation (12), and then promote trust. From the perspective of Social Capital, an excellent social network can help hospitals achieve better performance.

#### Proposition 2

The better the relationship between social network service innovation and the team, the trust of the team will also increase.

### Team Trust and Team Performance

The development of online platforms has triggered a new e-commerce model called social commerce. Use online platforms for social interaction to promote online purchases and sales of various products and services. In recent years, this development has involved many transaction-related issues. Trust is built on “social identification”. When the social identification information between an hospital and an hospital is similar, the presumptive trust between the two parties is stronger (11). Reputation, scale, information quality, transaction security, communication, and word of mouth are the key factors that determine the quality of an online platform. Therefore, the social identification information of the hospital is aggregated from the main characteristics of the members of the hospital. At the same time, the display of the leader's leadership can determine the appearance of the hospital, and it will also be presented in the hospital's social information. Consumers' trust has become a key factor in the success of commercial organizations. Leaders are very particular about product quality, which is reflected in the hospital's social recognition. Information may be on the excellent reputation of the hospital's products. For example, a trust relationship is established when both hospitals can clearly recognize their work role (work role) and take actions that match the role, so as to establish a presumed trust relationship in the exchange relationship (11). Therefore, the following proposition can be derived.

#### Proposition 3

The better the trust relationship between teams, the better the team's work performance.

### The Through Mechanism of Team Trust on Team Network Services and Team Performance

The emergence of the Internet platform brings different thinking directions for hospitals and consumers, and the added value created by mutual trust and reasonable and even distribution. The development of trust is crucial to task performance, and performance becomes an indicator of the degree of trust development. In the online platform market, although the innovation driven by product recognition innovation and differentiated services is felt, in the evolution and development of Taiwan's online platform, the innovative services of the online platform are gradually gaining the trust of consumers. The network platform provides more information services to meet the needs of consumers, and provides new services in the cloud through the information management system, which makes the convenience and stability of consumers' use relatively improved, and overcomes mobile devices. However, the network platform cloud service allows suppliers to expand to support a variety of online services and enhance many competitive advantages. For example: Internet platform and international SiteMinder link Online Travel Agent. From the current order operation mode, the original inventory adjustment, price adjustment, and switch room adjustment must be operated from the OTA, and the order creation method must be established manually. The order creation cycle must be established at any time, and the cancellation of the order to supplement the room must also be manually supplemented, and the new cloud service is provided through the information management system. The future operation mode of inventory adjustment, price adjustment, and switch room adjustment will be unified by the SiteMinder system. The order construction method is automatically transferred in. The order creation cycle is once every 3 min. The cancellation of the order and the replacement of the room are automatically replaced by the system operation. Therefore, the suppliers through the stakeholder relation are considered to be a powerful source of knowledge, because they often learn more Technology development, and because of these cooperative stakeholder relations and cooperation to develop new services, they also share investment and risks. On another level, it also has a considerable impact on hospital performance. In the network environment, when there is a partnership between hospitals, or cooperation between projects, which need to be shared or authorized among multiple hospitals, trust relationship can be used to establish trust between hospitals. Under the environment, it may be necessary to exchange and share resources between different domains. At this time, a role is required to serve as the information exchange role in the network environment of different hospitals. Therefore, an intermediary is established between multiple domains. The role of is to trust the hospital and share information. Therefore, the following proposition can be derived.

#### Proposition 4

The higher the trust between teams, the better the quality of network service innovation and team work performance.

### Moderating Relationship Between Hospitalal Agency Relationship and Team Network Service Innovation and Team Trust

People are increasingly realizing that value creation occurs in any hospital that produces something, and it becomes an input to the process in the business network. Innovation, network, and service are the key themes related to the processes related to service innovation in the supply network from a multidisciplinary perspective. Each topic provides the basic principles of interrelationship. From Schumpeter (1939, 1943) believes that innovation provides opportunities for prosperity. The network plays a key role in innovation and knowledge transfer. In addition, the significant growth of the global service industry, the innovation process in the supply network, is the internal interaction of the service sector An important part of our business network, which contributes to innovation in the supply chain (28).

The network of European regulators in industries such as telecommunications, securities, energy and transportation in Europe is considered to be an important example of European network governance law. From the point of view of principal-agent, coordination issues are the main factor that promotes the establishment of a network of regulatory agencies. The network is given a wide range of tasks and membership, but it has almost no formal powers or resources. Therefore, in terms of institutions, the spread of network governance is actually limited (29). According to (30), raise the question of social theory. That is, the question of structured agency, to what extent can it reflect the mutual penetration of agency and structure in social life. In an environment where the hospital is fully authorized to act as an agent, team network service innovation can be supported by various measures that enable the network hospital to progress, resulting in the promotion of high-impact team trust. Therefore, the following proposition is derived.

#### Proposition 5

The better the hospital agent relationship is, the better the relationship between team network service innovation and team trust will be enhanced.

When the agency relationship is lower, the relationship between team network service innovation and team trust will be lower.

**Figure 1:**
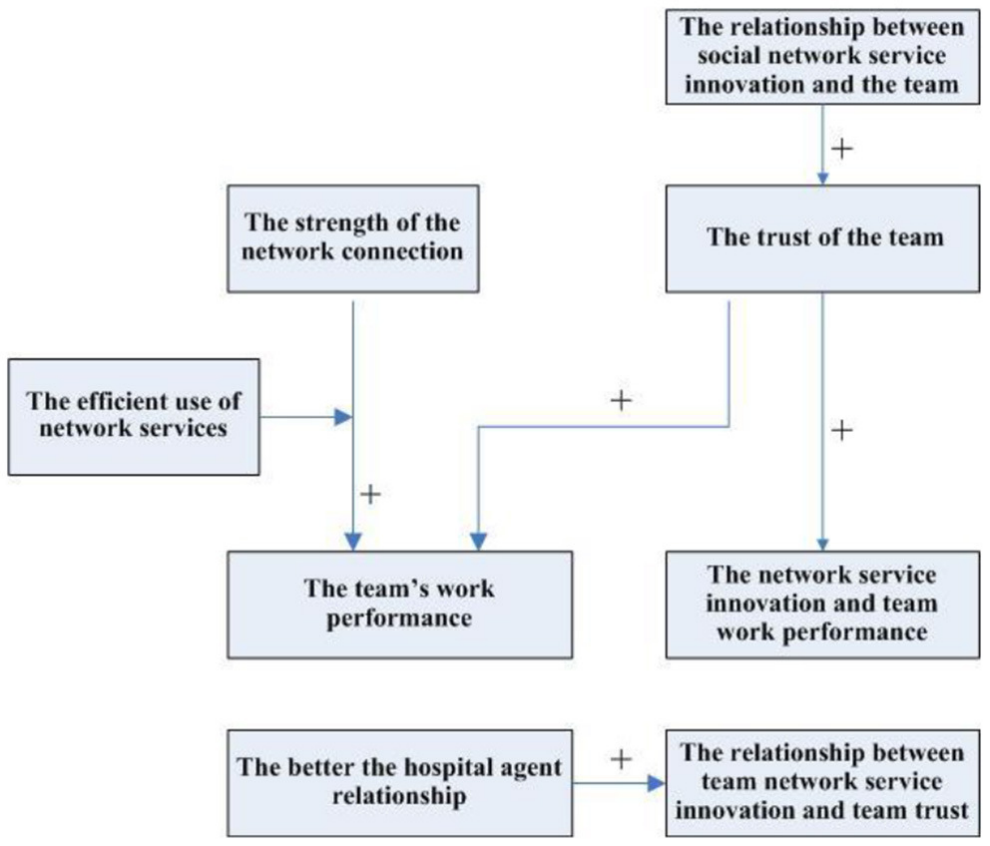
Relationship model between main factors.

According to the above propositions 1–5, the relationship between service innovation, inter-organizational trust, social network, agency and performance in hospital platforms can be obtained, as shown in Figure 1.

## Discussion and Conclusion

Cooperation and innovation are the keys to the survival of an organization. Firstly, trust between organizations is a necessary condition for organizational cooperation, and it is also the basis for establishing social networks between organizations. Secondly, because organizations trust each other, the innovative activities of organizations can be developed. Through the social network to exchange resources and opportunities, so that each organization in the network can obtain performance improvements. Finally, agency is a further demonstration of inter-organizational trust, which can be used to supplement the organization's own deficiencies and discover opportunities. Through the above discussion, this research discusses the important role of trust in organizational performance through two viewpoints: (Social Network) and (Agency Theory).

This study puts forward the above five propositions and proposes three future research directions based on the propositions: First, whether the relationship between the agent and the agent will affect the strength of the network connection, the willingness to cooperate and the willingness of the agent, and the efficiency of the entire network. Secondly, whether the reputation of the agent's past performance represents that his future performance will be good for the hospital, and whether the reputation can represent future performance, this point can be further explored in the future. Finally, if the trust relationship between the teams is not good, the relationship between the two hospitals is not good, whether it will affect the efficiency of the entire network. This is something that future research can explore.

## Data Availability Statement

The original contributions presented in the study are included in the article/supplementary material, further inquiries can be directed to the corresponding author.

## Author Contributions

All authors listed have made a substantial, direct, and intellectual contribution to the work and approved it for publication.

## Funding

Central Guidance on Local Science and Technology Development Fund of Sichuan Province (2021ZYD0156).

## Conflict of Interest

The authors declare that the research was conducted in the absence of any commercial or financial relationships that could be construed as a potential conflict of interest.

## Publisher's Note

All claims expressed in this article are solely those of the authors and do not necessarily represent those of their affiliated organizations, or those of the publisher, the editors and the reviewers. Any product that may be evaluated in this article, or claim that may be made by its manufacturer, is not guaranteed or endorsed by the publisher.
